# Vendor effects on murine gut microbiota and its influence on lipopolysaccharide-induced lung inflammation and Gram-negative pneumonia

**DOI:** 10.1186/s40635-020-00336-w

**Published:** 2020-08-25

**Authors:** Nora S. Wolff, Max C. Jacobs, Bastiaan W. Haak, Joris J. T. H. Roelofs, Alex F. de Vos, Floor Hugenholtz, W. Joost Wiersinga

**Affiliations:** 1 Center for Experimental and Molecular Medicine, Amsterdam Infection & Immunity Institute, Amsterdam UMC, location AMC, University of Amsterdam, Meibergdreef 9, 1105 AZ Amsterdam, Netherlands; 2 Department of Pathology, Amsterdam Cardiovascular Sciences, Amsterdam UMC, location AMC, University of Amsterdam, Meibergdreef 9, 1105 AZ Amsterdam, Netherlands; 3 Department of Medicine, Division of Infectious Diseases, Amsterdam UMC, location AMC, University of Amsterdam, Meibergdreef 9, 1105 AZ Amsterdam, Netherlands

**Keywords:** Intestinal microbiota, Vendor, Lipopolysaccharide, *Klebsiella pneumoniae*, Pulmonary Inflammation, Infection, Pneumonia, Sepsis

## Abstract

**Background:**

The microbiome has emerged as an important player in the pathophysiology of a whole spectrum of diseases that affect the critically ill. We hypothesized that differences in microbiota composition across vendors can influence murine models of pulmonary lipopolysaccharide (LPS) inflammation and Gram-negative pneumonia.

**Methods:**

A multi-vendor approach was used with genetically similar mice derived from three different vendors (Janvier, Envigo, Charles River). This model was employed to study the effect on the host response to a pulmonary LPS challenge (1 μg *Klebsiella pneumoniae* LPS, intranasal), as well as experimental *K. pneumoniae* infection (ATCC43816**,** 1 × 10^4^ CFU, intranasal).

**Results:**

Gut microbiota analysis revealed profound intervendor differences in bacterial composition as shown by beta diversity and at various taxonomic levels. Tumor necrosis factor (TNF)-α and interleukin (IL)-6 release in lung and bronchoalveolar lavage fluid (BALF) were determined 6 and 24 h after intranasal treatment with LPS. No differences were found between the groups, with the exception for Envigo, showing a higher level of TNFα in lung and BALF at 6 h compared to Janvier and Charles River. In another set of experiments, mice from different vendors were subjected to a clinically relevant model of Gram-negative pneumonia (*K. pneumoniae*). At 12 and 36 h post-infection, no intervendor differences were found in bacterial dissemination, or TNFα and IL-6 levels in the lungs. In line, markers for organ failure did not differ between groups.

**Conclusions:**

Although there was a marked variation in the gut microbiota composition of mice from different vendors, the hypothesized impact on our models of pulmonary inflammation and severe pneumonia was limited. This is of significance for experimental settings, showing that differences in gut microbiota do not have to lead to differences in outcome.

## Introduction

The microbiome has emerged as an important player in the pathophysiology of a whole spectrum of diseases that affect the critically ill with an impact on metabolism, the development of organ failure, and the host defense against pathogens [1–5]. It is therefore no surprise that differences in gut microbiota can lead to changes in experimental outcomes. For instance, mice from different vendors have differences in their gut microbiota, which can lead to a different outcome in models of asthma and infection [6–10].

Villarino et al. [6] showed that mice from different vendors exhibit differential susceptibility to malaria. Mice from vendors with high levels of members of the Firmicutes phylum in their cecal bacterial communities were resistant to infection with *Plasmodium yoelli*, whereas mice derived from vendors that exhibited high levels of members of the Bacteroidetes and Proteobacteria phyla had an increased susceptibility. Furthermore, Hilbert et al. [7] showed that vendor effects on the gut microbiota influence the host response in a murine model of abdominal sepsis in which stool was injected intraperitoneally. In that study, the severity of illness was shown to be dependent on the vendor of the mice used for the fecal injections [7]. This can be especially alarming considering the fact that replications of studies across the world are often done with mice from different vendors or facilities, which can show significant differences in the composition of their gut microbiota [11].

This “vendor effect” is showing increasing popularity as a model for different gut microbiota, sometimes showing insight into which bacteria might play an important role in the pathogenesis of a given disease or syndrome [6–8]. However, whether vendor-associated differences in the gut microbiota also affects models of pulmonary lipopolysaccharide inflammation and Gram-negative pneumonia remains to be described. The significance of this question is further emphasized by the emerging insights on the so-called gut-lung axis in the host defense against pneumonia. Indeed, cross talk between the gut microbiome and the lung has been suggested to modulate the pulmonary host response against inflammation and both viral and bacterial infections [3, 12–17]. We hypothesized that the murine gut microbiota varies among three major suppliers of laboratory animals often used for pulmonary inflammation and infection models and that these variations influence the phenotype of lipopolysaccharide (LPS)-induced lung inflammation and Gram-negative pneumonia caused by *Klebsiella pneumoniae*.

## Methods

### Mice

Specific-pathogen-free C57BL/6J male mice with similar genetic backgrounds were ordered from Janvier (C57BL/6JRj, Saint Berthevin Cedex, France), Envigo Research Models and Services (C57BL/6JOlaHsd, Horst, Netherlands (previously Harlan)), and Charles River (C57BL/6J, Sulzfeld, Germany) and housed in groups in individually ventilated cages enriched with disposable rodent homes and nestling paper. All mice received their in-house vendor specific diets ad libitum for the full length of all experiments. Janvier mice received the Ssniff Mouse Breeding Diet, Envigo the Teklad 2018 Global 18% Protein Rodent Diet (irradiated 2918), and Charles River mice the VRF1 (P) diet from Special Diets Services. All mice arrived at the same time, and the experiments were performed within 2 weeks. All mice were housed at the Animal Research Institute AMC (ARIA) of our institution where they acclimatized for 1 week prior to the commencement of experiments, at which time the mice were between 9 and 10 weeks of age and in good health. Mice were assessed on their welfare (including posture and activity) throughout their stay at the facility by both the researchers and the animal care takers.

### Study design

Experimental groups consisted of 8 mice spread across 2–3 cages, the number of animals was determined through sample size calculations using previous data for 80% power and affect size 1.85, with a significance level of 0.05. Some mice fought before the inoculation and had to be taken out of the experiment. Pulmonary inflammation and infection was induced by intranasal inoculation of 1 μg lipopolysaccharides (LPS) from *Klebsiella pneumoniae* (Sigma, L4268), or 10^4^ colony forming units (CFU) of *K. pneumoniae* serotype 2 (ATCC 43816), dissolved in 50 μl phosphate-buffered saline as described [18, 19]. Time points and dosages were selected based on previous publications. Klebsiella infection showing a well-established lung infection 12 h post-infection and dissemination to other body sites at 36 post infection, after 2 days this dose becomes lethal. Furthermore, this dose of LPS has been shown to lead to well-established inflammation in the lung at 6 h post-inoculation and at 24 h the local inflammation is becoming less, this dose does have lethal outcome in mice [18–20]. This was done under a light sedation with 2–3% isoflurane in 100% O_2_ to ensure that the mice would calmly breathe in the fluids. Mice inoculated with LPS were euthanized at 6 and 24 h post-inoculation while mice infected with *K. pneumoniae* were euthanized at 12 and 36 h post-inoculation as described [18, 19].

Sample collection, processing, and assays can be found in the Supplemental Digital Content.

### Statistical analysis

Analyses were performed using GraphPad Prism 7 software. Significance was calculated using the Kruskal–Wallis one-way ANOVA with an uncorrected Dunn’s test for analyses between groups. *P* values < 0.05 were considered statistically significant.

## Results

### Vendor affects bacterial gut microbiota composition

**Fig. 1:**
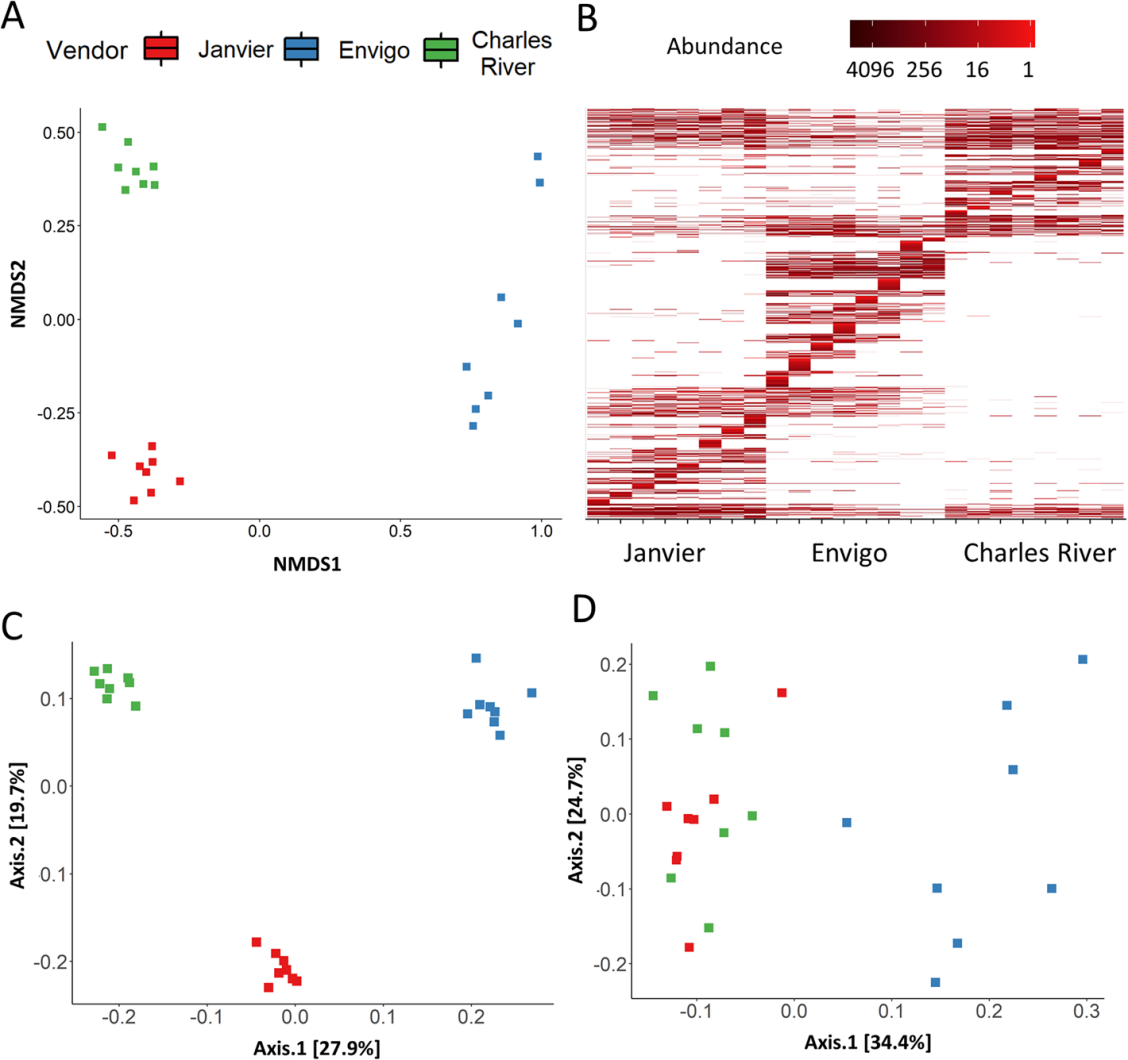
Beta diversity shows clear differences in gut microbiota composition between vendors. Fecal pellets were collected from mice from 3 different vendors (Janvier, Envigo, and Charles River) before intranasal inoculation with LPS or *K. pneumoniae*. **a** Bray-Curtis-based non-metric multidimensional scaling (NMDS) plot for all microbiome samples shows clearly separated clusters for each vendor. **b** Bray-Curtis NMDS heatmap in which the lines represent single amplicon sequence variants, in which a darker color correlates to a higher abundance. **c** Unweighted unifrac and weighted unifrac (**d**). The abbreviations used to indicate vendors are as follows: Janvier (Jan), Envigo (Env), and Charles River (CR), *n* = 8

**Fig. 2:**
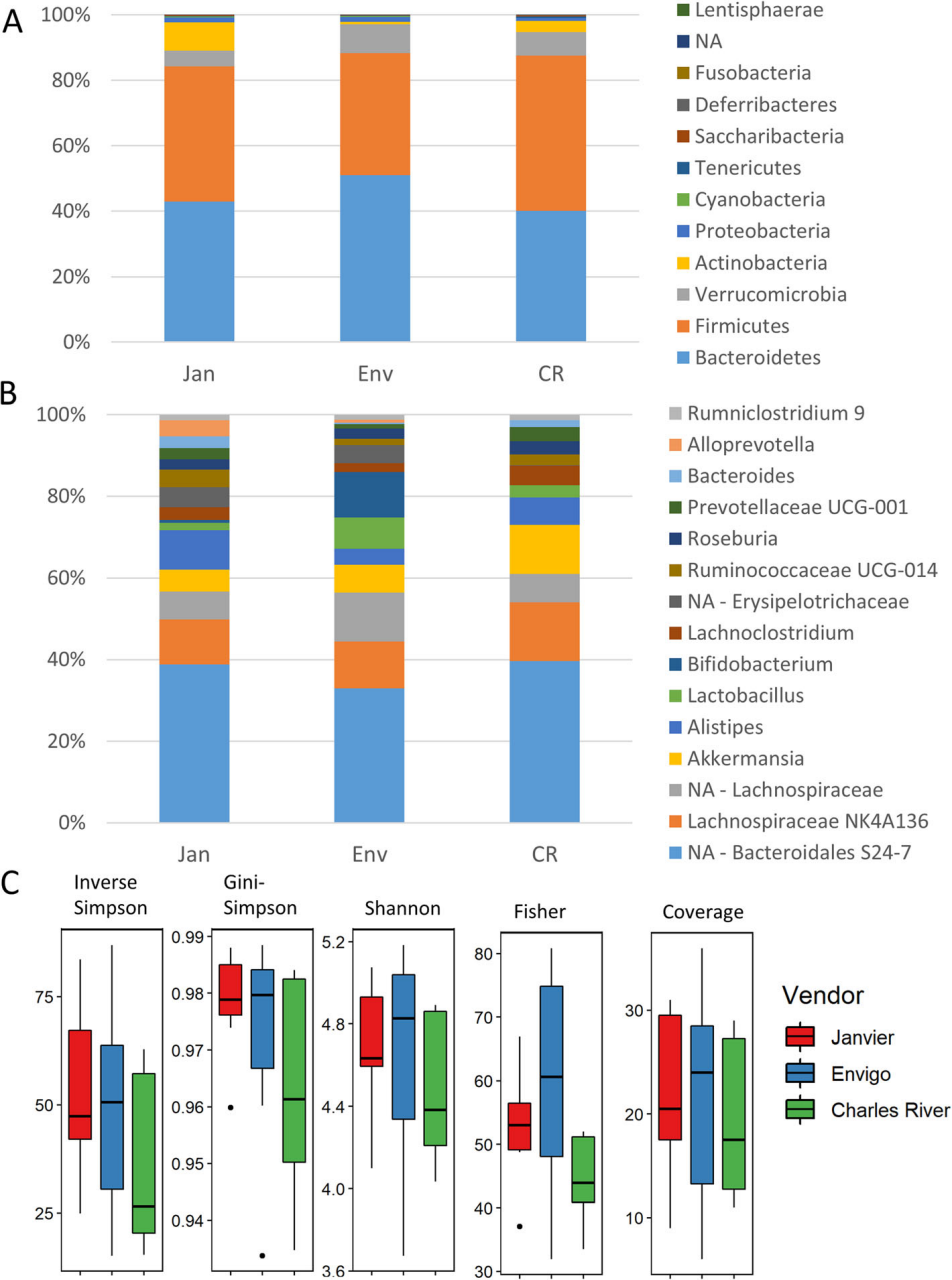
Alpha diversity and composition of gut microbiota. Fecal pellets were collected from mice from 3 different vendors (Janvier, Envigo, and Charles River) before intranasal inoculation with LPS or *K. pneumoniae*. **a** The presence of each phyla per vendor expressed in percentages. NA denotes non annotatable reads with unknown phylum classification. **b** The presence of the top 15 genera per vendor expressed in percentage, top 15 is based on abundance and makes 100%. **c** Alpha diversity, Inverse Simpson, Gini-Simpson, Shannon, Fisher, and Coverage. The abbreviations used to indicate vendors are as follows: Janvier (Jan), Envigo (Env), and Charles River (CR), *n* = 8

Genetically similar inbred strains of wild-type mice maintained by three major vendors (Janvier, Envigo, and Charles River) showed profound differences in their gut bacterial communities. Beta-diversity shows how different the microbial composition is from one vendor compared to another. Several beta-diversity analyses showed a distinct microbiota composition in each of the vendors, in both Bray-Curtis and weighted Unifrac analyses (Fig. 1a–c), although unweighted Unifrac showed an overlap in microbiota composition of Charles River and Janvier mice (Fig. 1d). Clear vendor differences were also seen at the phylum level (Fig. 2a and S1), most notably, the difference in the ratio of Firmicutes/Bacteroidetes where Envigo mice had a significantly lower ratio than mice from Janvier (*P* < 0.05). This was mainly due to differences in the abundance of Bacteroidetes (*P* < 0.01), as there was no significant difference in the abundance of Firmicutes. Furthermore, Actinobacteria were hardly present in Envigo mice and therefore significantly lower when compared to levels found in mice from Janvier (*P* < 0.05) (Fig. S1). There were also clear differences observed at the genus level (Fig. 2b and S2), including a low abundance of the genus Bacteroides in Envigo mice in comparison to Janvier (*P* < 0.001) and Charles River mice (*P* < 0.01), whereas the genus Bifidobacterium was found at higher abundances for mice from Envigo versus Janvier and Charles River mice (*P* < 0.05 and *P* < 0.01, respectively). Furthermore, the genus Alloprevotella was largely absent in mice from Charles River, whereas these were found in the other vendors (Fig. S2). Alpha diversity, a measure of species diversity between the microbial communities in the gut, was not different between vendors (Fig. 2c). Taken together, these data demonstrate that there was a vendor specific difference in gut microbiota composition between these genetically similar mice.

### Effect of vendor on LPS-induced lung inflammation

**Fig. 3:**
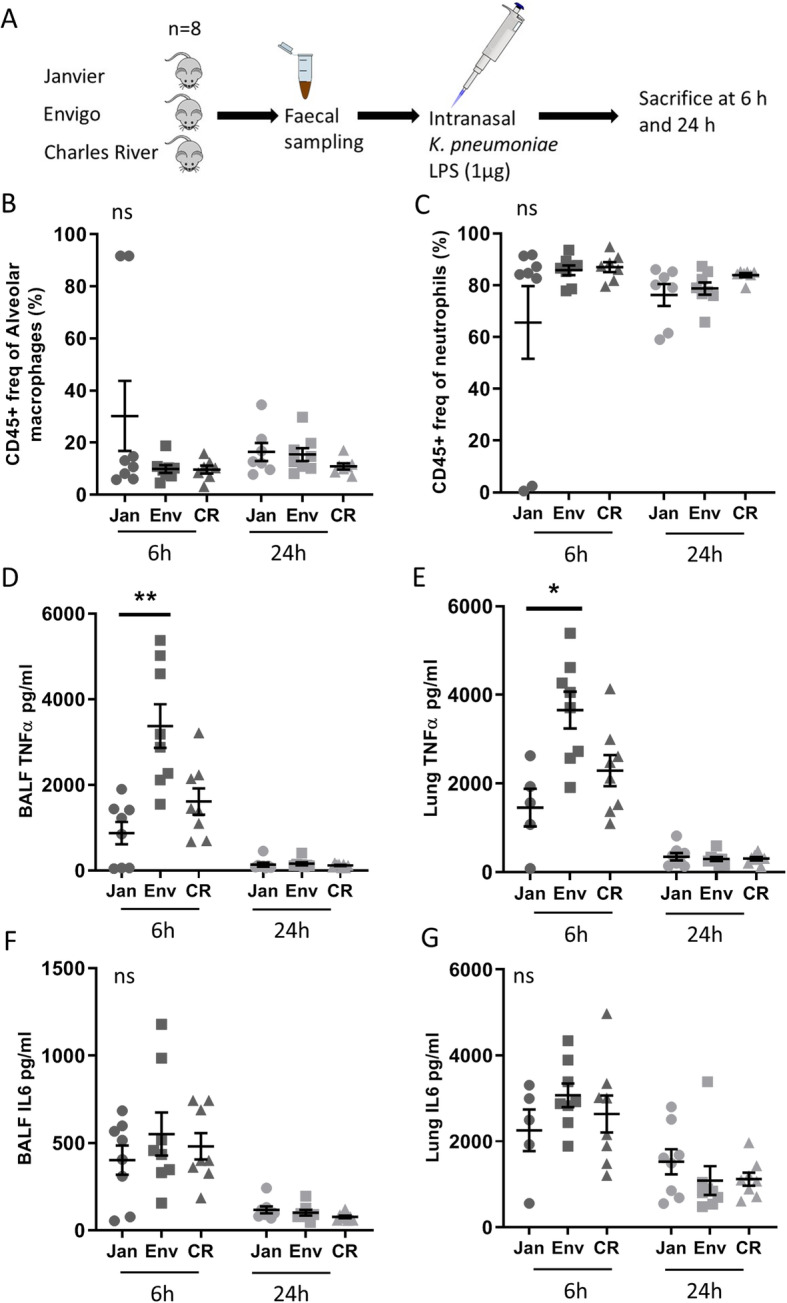
Experimental model of LPS inoculation and following cytokine and cell response. **a** Experimental design. Fecal samples were obtained from mice from 3 different vendors (Janvier, Envigo, and Charles River) prior to intranasal inoculation with 1 μg lipopolysaccharide (LPS) from *K. pneumoniae*. Mice were sacrificed at 6 and 24 h post-inoculation. Bronchoalveolar lavage fluid (BALF) was analyzed using flow cytometry. The graphs show the percentages of CD45-positive cells that are alveolar macrophages (**a**) or neutrophils (**b**). Tumor necrosis factor alpha (TNFα) (**d**, **e**) and interleukin 6 (IL-6) (**f**, **g**) in lung homogenate (**e**, **g**) and bronchoalveolar lavage fluid (BALF) (**d**, **f**) at 6 h and 24 h post-inoculation. The abbreviations used to indicate vendors are as follows: Janvier (Jan), Envigo (Env), and Charles River (CR). Results are shown as mean ± s.e.m. (*n* = 5–8), and ns denotes not significant. *p* < 0.05 (*) and *p* < 0.01 (**)

In order to test whether these microbiota differences have an impact on the murine response to pulmonary inflammation we first made use of a sterile lipopolysaccharide (LPS) model (Fig. 3a). Mice derived from different vendors received equal amounts of intranasal administered *Klebsiella pneumoniae* LPS. BALF and whole lung samples were obtained both 6 and 24 h after LPS administration, since these time points are representative for both polymorphonuclear cell influx and local cytokine/chemokine release [18, 21, 22]. Bodyweights between mice before inoculation were largely similar and showed no difference between vendors when comparing the weight change during LPS-induced inflammation (Fig. S3, A and B). LPS instillation resulted in a strong pulmonary influx of inflammatory cells. However, no intervendor differences where seen in the percentages of neutrophils or alveolar macrophages at both 6 and 24 h post-LPS challenge (Fig. 3b, c and S4). In all mice, LPS instillation induced a strong tumor necrosis factor alpha (TNFα) and interleukin 6 (IL-6) response, most prominently at 6 h post-inoculation (Fig. 3d–g). No differences were observed between vendors in terms of IL-6 response in both the lung and BALF (Fig. 3f, g). However, both BALF and lung TNFα levels were significantly higher at 6 h post-inoculation in Envigo mice when compared to the other vendors (only significant to Janvier *P* < 0.001 and *P* < 0.01, respectively). This difference disappeared at 24 h post-LPS treatment (Fig. 3d, e). Furthermore, additional cytokines and chemokines were measured in BALF and lung, including IL-1β, IL-10, and keratinocyte-derived chemokine (KC), showing no significant differences, except for the levels of KC in the lung in mice from Janvier and Envigo at 24 h post-inoculation (*P* < 0.05) (Table S1). Taken together, these data show a significant vendor specific difference in the early TNFα response during sterile pulmonary inflammation.

### Vendor differences do not impact the host response during *K. pneumoniae* pneumonia

**Fig. 4:**
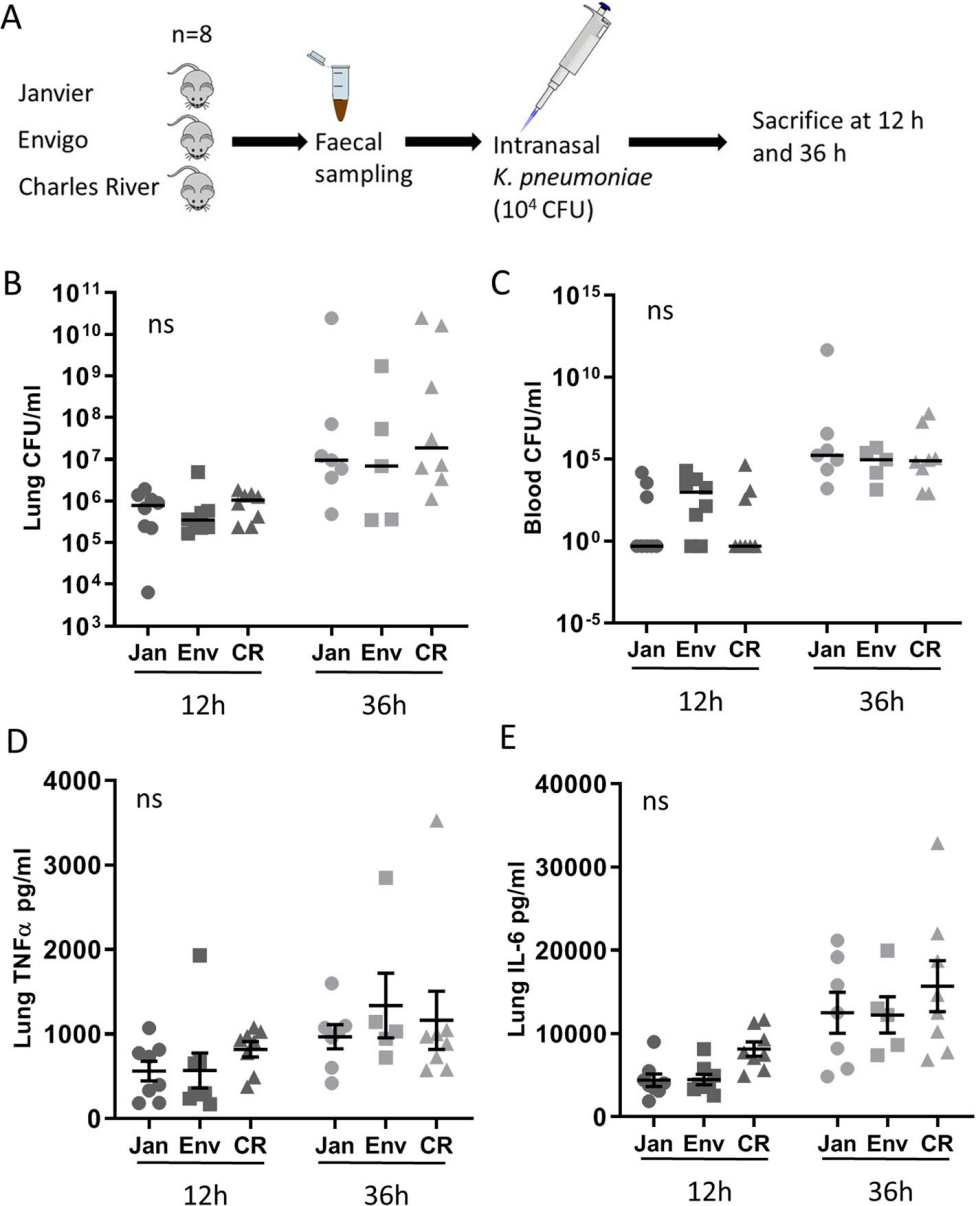
Vendor associated gut microbiota does not influence host response against *K. pneumoniae* infection. **a** Experimental design. Fecal samples were obtained from mice from 3 different vendors (Janvier, Envigo, and Charles River) prior to intranasal inoculation with 10^4^ colony-forming units (CFU) of *K. pneumoniae* (*n* = 5–8 per group). Mice were sacrificed at 12 h and 36 h post-infection. Pulmonary (**b**) and blood (**c**) colony-forming units (CFU) at 12 h and 36 h post-infection. Tumor necrosis factor alpha (TNFα) (**d**) and interleukin 6 (IL-6) (**e**) levels in lung homogenates at 12 h and 36 h post-infection. The abbreviations used to indicate vendors are as follows: Janvier (Jan), Envigo (Env), and Charles River (CR). Data is shown as median (CFU) or mean ± s.e.m. (Cytokines), and *n* = 8, ns denotes not significant

To investigate whether intervendor differences could have an impact on the host response against pulmonary infection, we inoculated mice intranasally with 10^4^ CFU viable *K. pneumoniae* and harvested lung, blood, and liver at predefined time points for quantitative cultures, seeking to collect data representative for local defense, at the primary site of infection, and subsequent dissemination (Fig. 4a) [19, 20]. There were some significant differences in weight in between the vendors prior to inoculation; however, there were no significant differences between vendors when comparing weight change during infection (Fig. S3, C and D). Bacterial counts in blood and homogenates of lung and liver showed no significant differences between vendors at all time points (Fig. 4b, c and S5A). To study the influence of vendor effects on cytokine release in Gram-negative pneumonia, we measured the levels of TNFα and IL-6 in lung homogenates harvested from mice after intranasal infection with *K. pneumoniae*. TNFα and IL-6 levels in lung homogenates did not show any intervendor differences at both time points (Fig. 4d, e). Further cytokine/chemokine analyses were conducted on blood plasma and lung including IL-6, TNF, monocyte chemotactic protein-1 (MCP-1) and interferon (IFN)-γ, IL-1β, IL-10, and KC, showing no significant differences except for IL-6 levels in blood plasma of Janvier and Envigo mice at 12 h (*P* < 0.05), which was not found at 36 h (Additional file 1: Table S2).

**Fig. 5:**
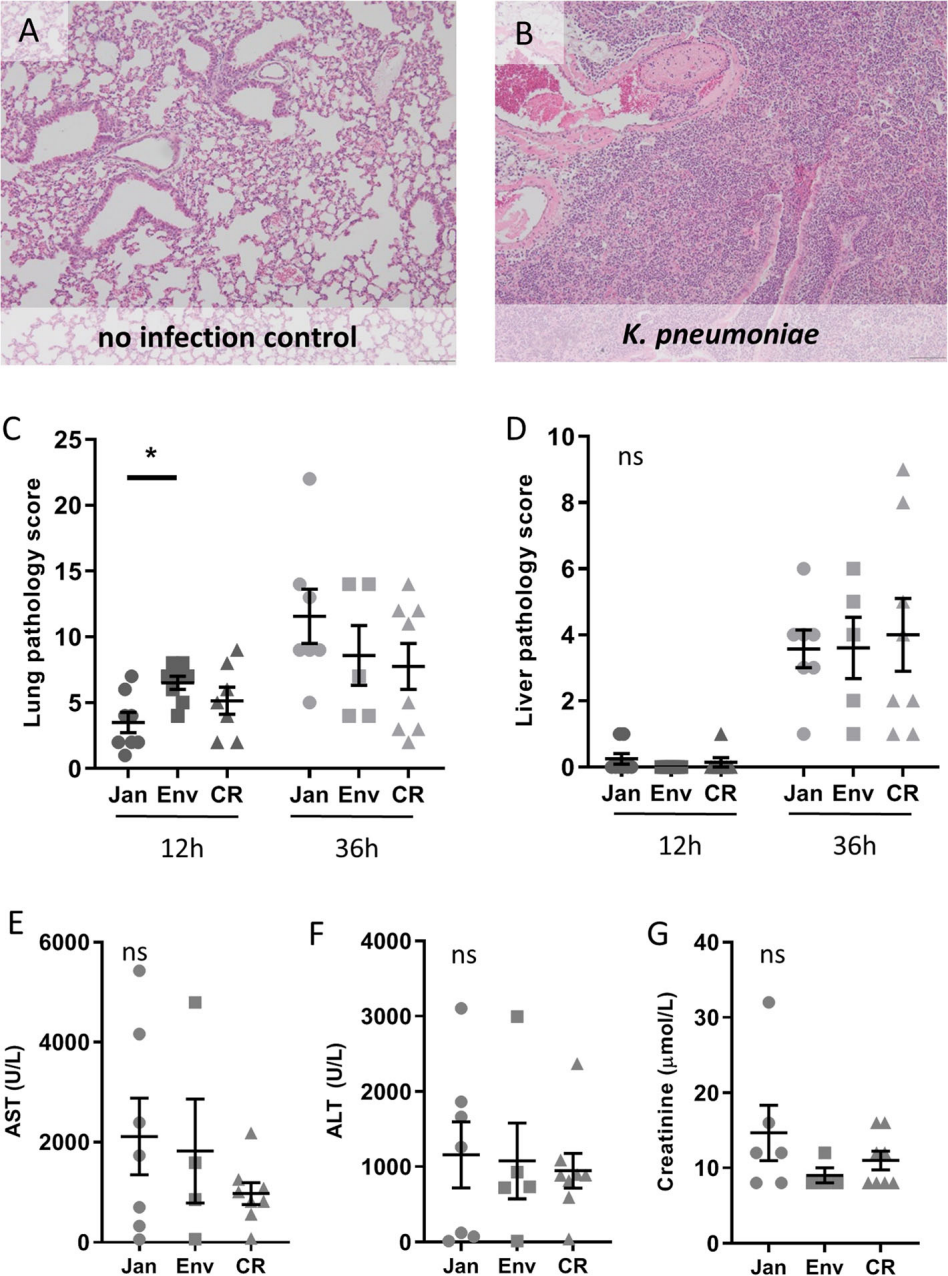
Influence of vendor on organ damage during *K. pneumoniae* infection. Representative microphotographs of lung tissue from uninfected (**a**) and 36 h infected with *K. pneumoniae* (**b**), showing bronchitis, confluent alveolar inflammation (pneumonia), thrombi, and perivascular edema (H&E staining, × 10 magnification). Pathology scores of lung (**c**) and liver (**d**) were calculated as described in the “Methods” section. Blood plasma from 36 h post-infection was used to measure aspartate aminotransferase (AST; **e**) and alanine aminotransferase (ALT; **f**), both parameters of liver cell damage, as well as creatinine (**g**), a parameter for kidney function. The abbreviations used to indicate vendors are as follows: Janvier (Jan), Envigo (Env), and Charles River (CR). Results are shown as mean ± s.e.m. (*n* = 5–8), and ns denotes not significant, *p* < 0.05 (*)

To further obtain insight into a potential vendor effect on the inflammatory response following *K. pneumoniae* infection, we semi-quantitatively scored lung and liver tissue sections generated 12 and 32 h after infection. Infected lung tissue showed overt signs of severe bronchopneumonia, characterized by bronchitis, areas of confluent parenchymal inflammation, presence of thrombi and edema (Fig. 5a, b). The pathology score of the lung only showed an early difference in inflammation between the Envigo and Janvier mice (*P* < 0.05), but no differences were present between the other groups nor at the later time point (Fig. 5c). In line, no differences between groups were seen in the liver pathology (Fig. 5d). Since neutrophil recruitment to the lung is an essential part of the inflammatory host response to pneumonia, we determined the granulocyte influx into the pulmonary compartment by lymphocyte antigen 6 complex, locus G (Ly-6G) immunostaining. No inter vendor differences were observed (Fig. S5B). Liver and kidney function/damage was measured through markers in blood plasma at 36 h post-infection when the bacteria have disseminated from the lung towards distant organs. Aspartate aminotranspherase (AST), alanine aminotranspherase (ALT), and creatinine levels all showed no significant differences between vendors (Fig. 5e, f). These experiments suggest that intervendor differences do not have a major influence on the host response in this model of *K. pneumoniae*-induced pneumonia.

## Discussion

This study demonstrates that genetically similar C57BL/6J mice from different vendors can have significant differences in their gut microbiota. However, these differences did not translate in large effects on the pulmonary host response during sterile or bacterial inflammation. More specifically, only limited vendor-associated differences were found in the early TNFα response during LPS-induced lung inflammation and the early lung pathology scores during *K. pneumoniae* infection. However, other measured parameters, including bacterial loads in the lung, bacterial dissemination towards distant organs, pulmonary levels of IL-6, and neutrophil influx in the lung during inflammation and infection, did not show a vendor effect in these experimental models.

These largely negative results were initially unexpected as previous studies have showed that microbiome-associated vendor effects can influence outcome in murine models of infection and asthma [6–10]. Villarino et al. [6] used mice from different vendors for an experimental *Plasmodium yoelli* infection. The vendor-effect they saw led to the finding that treatment of mice with Lactobacillus and Bifidobacterium can reduce the parasite burden during *Plasmodium yoelli* infection. This is an elegant example of how the multi-vendor approach was used as a model to investigate the involvement of the gut microbiota in disease, an approach which is gaining popularity. Furthermore, there is an increased awareness on the influence of the microbiome on the outcome of experimental models of infection and inflammation [6–10, 12, 15]. In addition, it should be realized that not only vendor but also diet, environment, and administered drugs can all affect the composition of the microbiome [11, 23–25]. Therefore, it is very interesting that we did find clear differences in the gut microbiota, but very limited vendor-associated effects in experimental murine models of LPS induced in lung inflammation and *K. pneumoniae* pneumonia.

A recent study from Rosshart et al. [26] highlighted the difference in gut microbiota between laboratory mice and wild mice. The authors used a model in which they created so-called “wildlings” by transferring C57BL/6 embryos into wild mice. These wildlings were shown to genetically be like the laboratory mice, but that their bacterial microbiome resembled that of wild mice (skin, gut, and vaginal). Furthermore, these differences had an impact on the immune landscape of the spleen and blood. They even showed some examples where these wildling mice were better at predicting human clinical outcomes than laboratory mice. This information together with vendor studies show how big of an impact differences in environment/diet can have on experimental outcomes, suggesting that we might need a new approach/view to the gut microbiota in research settings.

The intestinal microbiota has been shown to play key roles in both local and systemic immunity. Recent works into the intestinal microbiota are starting to show an association between the composition of the gut microbiota and lung health, also known as the gut-lung axis [3, 12, 27–29]. The depletion of the gut microbiota with antibiotics has been shown to lead to worsened outcomes in experimental models of infection with *Streptococcus pneumoniae*, *K. pneumoniae, Burkholderia pseudomallei*, and *influenza* [12–15, 30]*.* Several mechanisms have been proposed on how the gut microbiome can influence lung immunity. Dietary fibers are metabolized by the gut microbiota, which will result in increased concentrations of circulating short-chain fatty acids (SCFAs) as well as an altered Firmicutes/Bacteroidetes ratio [31]. Trompette et al. [31] showed that mice fed high fermentable fiber diets were protected against allergic inflammation in the lung, whereas mice that were fed with low fiber diets showed increased allergic airway disease [31]. Furthermore, the exposure of NOD-like receptors and Toll-like receptor agonists in the intestines by substances such as lipoteichoic acid and LPS have been demonstrated to increase the lungs’ ability to clear bacteria [28]. It is therefore an interesting finding that the microbiota differences that we observed only had a limited impact on the host response during a pulmonary challenge with LPS or *K. pneumoniae*.

This study has a number of limitations. Mice were genetically similar; however, they all originally came from the C57BL/6J lineage, but were inbred in different companies for many generations, they are therefore similar and not identical (shown by the addition of letters following C57BL/6J). Once the mice arrived in the facilities, they were kept on the diets that they received at their vendors; this is to prevent any microbiome shifts due to exposure to a new diet. Although these diets were similar, they were not identical which potentially influenced the gut microbiota composition. Furthermore, we did not investigate potential epigenetic differences between these mice. In addition, it should be acknowledged that another vendor, or another batch could have a different gut microbiota than what we encountered. Moreover, we used one strain of *K. pneumoniae* for our infection model, whereas different clinical isolates of *K. pneumoniae* have been shown to demonstrate a strain depend variation in innate immune response [32]. For this study, we did not use mortality as an endpoint to study vendor-related mortality differences; this choice was largely made to reduce the needed mice within a largely a negative study. It would also have been interesting to see whether these mice also had vendor-associated difference in the fungi and viruses present in the intestines. Finally, our study did not investigate potential differences in the composition of the lung microbiota between vendors. It remains to be established however if the lung microbiome plays any significant role during LPS inflammation or bacterial pneumonia.

## Conclusions

In conclusion, this study shows that there was a clear difference in the composition of gut microbiota of mice from different vendors. These vendor-specific differences in microbiome composition however only had a limited impact on the host response during pulmonary LPS inflammation and *K. pneumoniae*-induced pneumonia. These results were in contrast with our original hypothesis that these vendor-specific effects would influence the phenotype in these models. This is of significance for experimental setting, showing that differences in gut microbiota do not have to lead to differences in outcome.

## Supplementary information

**Additional file 1: Table S1.** cytokine and chemokine levels in Lung and BALF following intranasal LSP administration. Mice received an intranasal inoculation with 1 μg lipopolysaccharide (LPS) from K. pneumoniae, and were sacrificed at 6 and 24 h post inoculation. Results are shown as mean (s.e.m.), BDL = below detection limit, ns= not significant, *n*=5-8. **Table S2.** cytokine and chemokine levels in plasma and Lung following intranasal infection of *K. pneumoniae*. Mice received an intranasal infection with 10.000 CFU *K. pneumoniae*, and were sacrificed at 12 and 36 h post inoculation. Results are shown as mean (s.e.m.), BDL = below detection limit, ns= not significant, *n*=8.

**Additional file 2: Supplemental Figure 1.** Phylum abundance of gut microbiota between vendors. Graphs show the reads per vendor for each phylum separately, NA denotes non annotatable reads with unknown phylum classification (A), and the ratio of Firmicutes/Bacteroidetes (B). Of note, only Bacteroidetes and Deferribacteres had *P*<0.05 in the Kruskal-Wallis analysis. The abbreviations used to indicate vendors are as follows: Janvier (Jan), Envigo (Env) and Charles River (CR). Results are shown as mean ± s.e.m. (*n*= 8), ns denotes not significant, *P*<0.05 (*), *P*< 0.01 (**), ● denotes adjusted *p*-value <0.05 using the Benjamini and Hochberg analysis.

**Additional file 3: Supplemental Figure 2.** Genera abundance of the top 15 gut microbiota between vendors. Graphs show the reads per vendor for the top 15 genera (based on abundance), one graph per genus. The abbreviations used to indicate vendors are as follows: Janvier (Jan), Envigo (Env) and Charles River (CR). Results are shown as mean ± s.e.m. (*n*= 8), ns denotes not significant, *P*<0.05 (*), *P*< 0.01 (**), *P*<0.001 (***), *P*<0.0001 (****), ● denotes adjusted *p*-value <0.05 using the Benjamini and Hochberg analysis.

**Additional file 4: Supplemental Figure 3.** Murine weight prior to administration of LPS or *K. pneumoniae* and weight change during challenges. Mice were weight prior to inoculation and at sacrifice. The weight prior to administration of *K. pneumoniae* LPS (A) and *K. pneumoniae* (C). For these graphs the time points show the groups separated to the time at which they will be sacrificed after the infection/inflammation. The change in weight during *K. pneumoniae* LPS challenge (B) and *K. pneumoniae* infection (D). The abbreviations used to indicate vendors are as follows: Janvier (Jan), Envigo (Env) and Charles River (CR). Results are shown as mean ± s.e.m. (*n*=5-8), ns denotes not significant, *P*<0.05 (*), *P*< 0.01 (**), *P*<0.001 (***).

**Additional file 5: Supplemental Figure 4.** Flow cytometry gating strategy. Bronchoalveolar lavage fluid (BALF) was analyzed using flow cytometry, to determine the percentage of alveolar macrophages and neutrophils (from CD45 positive cells) after intranasal LPS (1 μg) administration.

**Additional file 6: Supplemental Figure 5.** Bacterial growth in liver and Ly-6G in lung upon *K. pneumoniae* infection. Bacterial colony forming units (CFU) of the liver at 12 h and 36 h post infection (A). Sections of lung were cut, stained and quantified for Ly-6G (see supplementary methods) (B). The abbreviations used to indicate vendors are as follows: Janvier (Jan), Envigo (Env) and Charles River (CR). Data is shown as median (CFU) or mean ± s.e.m. (Ly-6G), *n*=5-8, ns denotes not significant.

## Data Availability

The datasets used and/or analyzed during the current study are available from the corresponding author on reasonable request.

## Abbreviations

ALT: Alanine aminotranspherase
AST: Aspartate aminotranspherase
BALF: Bronchoalveolar lavage fluid
CR: Charles River
Env: Envigo
IL-10: Interleukin 10
IL-1β: Interleukin 1 beta
IL-6: Interleukin 6
Jan: Janvier
*K. pneumoniae*: *Klebsiella pneumoniae*
KC: Keratinocyte-derived chemokine
LPS: Lipopolysaccharide
Ly-6G: Lymphocyte antigen 6 complex, locus G
TNFα: Tumor necrosis factor alpha
