# Supplementary material for: Vendor effects on murine gut microbiota and its influence on lipopolysaccharide-induced lung inflammation and Gram-negative pneumonia
Source: Intensive Care Med Exp. 2020 Aug 25;8:47. doi: 10.1186/s40635-020-00336-w (PMC7447702; doi:10.1186/s40635-020-00336-w)
Supplement: Supplementary file 1 — Additional file 1: Table S1. cytokine and chemokine levels in Lung and BALF following intranasal LSP administration. Mice received an intranasal inoculation with 1 μg lipopolysaccharide (LPS) from K. pneumoniae, and were sacrificed at 6 and 24 h post inoculation. Results are shown as mean (s.e.m.), BDL = below detection limit, ns= not significant, n=5-8. Table S2. cytokine and chemokine levels in plasma and Lung following intranasal infection of K. pneumoniae. Mice received an intranasal infection with 10.000 CFU K. pneumoniae, and were sacrificed at 12 and 36 h post inoculation. Results are shown as mean (s.e.m.), BDL = below detection limit, ns= not significant, n=8. [file 40635_2020_336_MOESM1_ESM.docx]

**Supplemental Digital Content**

**Vendor effects on murine gut microbiota and its influence on lipopolysaccharide induced lung inflammation and Gram-negative pneumonia**

Nora S. Wolff, MSc, Max C. Jacobs, MSc, Bastiaan W. Haak, MD, Joris J. T. H. Roelofs, MD, PhD, Alex F. de Vos, PhD, Floor Hugenholtz, PhD, W. Joost Wiersinga, MD, PhD

**Supplemental methods**

**Sample collection and processing**

Experimental groups consisted of 8 male mice spread across 2-3 cages, the number of animals was determined through sample size calculations using previous data for 80% power and effect size 1.85, with a significance level of 0.05. Of all mice, fresh feces were collected just before inoculation, snap-frozen in liquid nitrogen and stored at -80 °C. Euthanasia was performed using an intraperitoneal injection of ketamine and dexmedetomidine followed by a cardiac puncture, of which the blood was collected in heparin and immediately cooled. Bronchoalveolar lavage fluid (BALF) was obtained in addition to the harvesting of lung and liver as described [1-4]. In short, lung and liver were submerged into 4% buffered formaldehyde for 24 hours followed by 48 hours in 70% ethanol before embedding in paraffin. Furthermore, different lung and liver samples were homogenized in isotonic saline (4 mL per gram of tissue). Bacterial load of the lung, liver and blood were determined by a dilution series plated onto sheep-blood agar plates and incubated for 16 hours at 37°C, after which the colony forming units (CFU) were counted. Following this, the blood was centrifuged at 3000 rpm for 10 minutes at 4°C, in order to obtain the blood plasma. BALF was centrifuged at 1250 RPM for 10 minutes at 4°C, the supernatant was used for following cytokine analysis and the cells for flow cytometry analysis. The lung and liver homogenates were diluted 1:1 with a lysis buffer (1% (v/v) Triton X-100,150 mM NaCl, 15 mM Tris, 1 mM MgCl(H_2_O)_6_, 1 mM CaCl_2_(H_2_O)_2_, Ph 7,4) including a protease inhibitor (Complete protease inhibitor cocktail tablets, Roche, Basel, Switzerland) and incubated on ice for 30 minutes, followed by centrifugation at 4000 rpm, for 10 minutes at 4°C, after which the supernatant was stored.

**Fecal microbiota analyses**

The fecal pellets were used for microbiota analysis. DNA was extracted using the double bead-beating protocol as described in protocol 5 of Costea *et. al.* [5], using S.T.A.R. buffer (Roche, Basel, Switzerland) instead of the described lysis buffer. The DNA was purified using the Maxwell® RSC Blood DNA Kit (Promega, Madison, USA). The DNA was PCR amplified using barcoded primers for the V3 and V4 regions of 16S rRNA as described [6]. For the purification of the amplified product, the AMPure XP beads (Beckman Coulter, Indianapolis, USA) were used according to manufacturer’s guidelines on a Beckman Coulter Biomex FX**.** The sequence was run on the Illumina MiSeq with the MiSeq V3 - 600 cycle kit from Illumina. The forwards and reverse reads were length trimmed at 240 and 210 respectively, which were inferred and merged with ASVs using DADA2 V.1.5.2 [7]. The assignment of taxonomy was done using the DADA2 implementation of the RDP classifier [8] and SILVA 16S [9]. Further processing was done using R (version 3.5.1), using several r packages, including phyloseq (version 1.27.6 [10]) and microbiome (1.4.2 [11]). To process the sequencing data the sequences were normalized to the smallest sample, which has 47,136 reads.

**Assays**

Interleukin (IL)-6, IL-1β, IL-10, keratinocyte-derived chemokine (KC) and tumor necrosis factor (TNF)-alpha levels were measured by enzyme-linked immunosorbent assays (ELISA) in accordance with manufacturers’ recommendation (R&D systems, Minneapolis, USA). In blood plasma, IL-6, TNF, monocyte chemotactic protein -1 (MCP-1) and interferon (IFN)-γ levels were determined by a cytometric bead array multiplex (the Mouse Inflammation Kit, BD Biosciences, New Jersey, USA). In order to quantify pulmonary influx of inflammatory cells, flow cytometry was performed as previously described [12], where alveolar macrophages are defined as CD45^+^/CD11c^+^/CD11b^-^, and neutrophils CD45^+^/CD11c^-^/LY-6G^+^. Flow cytometry was performed using a FACS CANTO II and using BD FACSDIVA^TM^ and FlowJo (LLC) software (all BD Biosciences, New Jersey, USA). BALF cells were resuspended in FACS buffer containing 5% BSA, 0.35 mM EDTA, 0.01% NaN_3_. Staining was performed following the manufacturer’s recommendations as described [12]. Aspartate aminotranspherase (AST), alanine aminotranspherase (ALT), and creatinine levels were measured using a c702 Roche Diagnostic machine.

**Histopathology**

Paraffin embedded lung and liver were cut into 4 µm thick sections and stained with hematoxylin and eosin. Slides were coded blinded by an experiences histo-pathologist in a blinded fashion. Lung tissue was graded for interstitial inflammation, endothelialitis, bronchitis, edema, pleuritis, and presence of thrombi, whereas liver tissue was graded for necrosis, parenchymal inflammation and presence of thrombi. The pathology score was the sum of all categories per organ, where each category was scored 0 to 4 where 0 is absent and 4 severe as described [2, 3]. Lymphocyte antigen 6 complex, locus G (Ly-6G) staining was performed using FITC-labelled rat anti-mouse ly-6G monoclonal antibody (127605, Biolegend, San Diego, USA), rabbit anti FITC (4510-7804, Bio-Rad, California, USA) and goat anti-rabbit HRP (DPVR-55 HRP, VWR, Pennsylvania, USA), after which the stained slides were digitizes using an automated slide scanner (Olympus, The Netherlands). Ly-6G staining was quantified using imageJ (version 2006.02.01, US National Institutes of Health Bethesda, MD) and expressed as percentage of total lung surface area.

**Outcome measurements**

Primary outcomes for the LPS challenge were the levels of TNFα in lung and BALF. Secondary outcomes were: levels of IL-6 in BALF and lung and the macrophages and neutrophils presence in BALF. The primary outcomes for *K. pneumoniae* infection were the CFU in lung, liver and blood. The secondary outcomes were: pulmonary levels of TNFα and IL-6, lung pathology score and Ly-6G staining, plasma markers for kidney and liver function/damage (AST, ALT, and creatinine).

**Supplemental tables**

**Table S1: cytokine and chemokine levels in Lung and BALF following intranasal LSP administration.**

|  | **Jan** | **Env** | **CR** | **significance** |
| --- | --- | --- | --- | --- |
| **Lung** |  |  |  |  |
| **IL-1β**  6h  24h | 957.0 (266.9)  239.7 (47.13) | 1476 (216.6)  156.9 (22.24) | 908.2 (215.6)  140.3 (14.74) | ns  ns |
| **IL-10**  6h  24h | 5290 (1318)  1651 (602.0) | 5885 (995.1)  486.2 (101.0) | 4251 (1122)  260.9 (76.97) | ns  ns |
| **KC**  6h  24h | 3806 (891.0)  4890 (822.8) | 5322 (351.7)  2331 (708.0) | 5444 (777.8)  3364 (716.5) | ns  * Jan- Env (p=0.0133) |
| **BALF** |  |  |  |  |
| **IL-1β**  6h  24h | BDL  BDL | BDL  BDL | BDL  BDL |  |
| **IL-10**  6h  24h | BDL  BDL | BDL  BDL | BDL  BDL |  |
| **KC**  6h  24h | 318.5 (68.71)  229.2 (44.77) | 398.0 (48.87)  208.9 (50.87) | 336.5 (36.92)  188.5 (24.44) | ns  ns |

Mice received an intranasal inoculation with 1 µg lipopolysaccharide (LPS) from K. pneumoniae, and were sacrificed at 6 and 24 h post inoculation. Results are shown as mean (s.e.m.), BDL = below detection limit, ns= not significant, n=5-8.

**Table S2: cytokine and chemokine levels in plasma and Lung following intranasal infection of *K. pneumoniae*.**

|  | **Jan** | **Env** | **CR** | **significance** |
| --- | --- | --- | --- | --- |
| **Plasma** |  |  |  |  |
| **IL-6**  12h  36h | 187.9 (72.59)  332.0 (94.72) | 99.13 (32.16)  696.6 (239.7) | 333.2 (110.8)  553.0 (238.8) | * Jan-Env (P=0.0300)  ns |
| **TNF**  12h  36h | 87.68 (53.34)  103.6 (18.38) | 28.67 (10.83)  153.9 (79.25) | 53.39 (41.16)  100.2 (24.40) | ns  ns |
| **MCP-1**  12h  36h | 912.4 (447.1)  1092 (245.5) | 694.6 (187.0)  2582 (1038) | 336.2 (51.78)  1912 (610.8) | ns  ns |
| **IFNγ**  12h  36h | BDL  22.78 (8.58) | BDL  39.09 (11.40) | BDL  25.01 (9.70) | ns |
| **Lung** |  |  |  |  |
| **IL-1β**  12h  36h | 160.8 (49.86)  1386 (189.9) | 125.3 (26.19)  1998 (345.7) | 285.0 (51.78)  1628 (220.4) | ns  ns |
| **IL-10**  12h  36h | 2381 (698.4)  6126 (724.4) | 2174 (334.4)  8025 (1075) | 3513 (924.5)  5266 (551) | ns  ns |
| **KC**  12h  36h | 7891 (1197)  13948 (3941) | 8312 (901.4)  12432 (1500) | 10127 (1333)  14830 (2698) | ns  ns |

Mice received an intranasal infection with 10.000 CFU *K. pneumoniae*, and were sacrificed at 12 and 36 h post inoculation. Results are shown as mean (s.e.m.), BDL = below detection limit, ns= not significant, n=8.

**Supplemental figure legends**

**Supplemental Figure 1. Phylum abundance of gut microbiota between vendors.** Graphs show the reads per vendor for each phylum separately, NA denotes non annotatable reads with unknown phylum classification (**A**), and the ratio of Firmicutes/Bacteroidetes (**B**). Of note, only Bacteroidetes and Deferribacteres had P<0.05 in the Kruskal-Wallis analysis. The abbreviations used to indicate vendors are as follows: Janvier (Jan), Envigo (Env) and Charles River (CR). Results are shown as mean ± s.e.m. (n= 8), ns denotes not significant, P<0.05 (*), P< 0.01 (**), ● denotes adjusted p-value <0.05 using the Benjamini and Hochberg analysis.

**Supplemental Figure 2. Genera abundance of the top 15 gut microbiota between vendors.** Graphs show the reads per vendor for the top 15 genera (based on abundance), one graph per genus. The abbreviations used to indicate vendors are as follows: Janvier (Jan), Envigo (Env) and Charles River (CR). Results are shown as mean ± s.e.m. (n= 8), ns denotes not significant, P<0.05 (*), P< 0.01 (**), P<0.001 (***), P<0.0001 (****), ● denotes adjusted p-value <0.05 using the Benjamini and Hochberg analysis.

**Supplemental Figure 3. Murine weight prior to administration of LPS or *K. pneumoniae* and weight change during challenges.** Mice were weight prior to inoculation and at sacrifice. The weight prior to administration of *K. pneumoniae* LPS (**A**) and *K. pneumoniae* (**C**). For these graphs the time points show the groups separated to the time at which they will be sacrificed after the infection/inflammation. The change in weight during *K. pneumoniae* LPS challenge (**B**) and *K. pneumoniae* infection (**D**). The abbreviations used to indicate vendors are as follows: Janvier (Jan), Envigo (Env) and Charles River (CR). Results are shown as mean ± s.e.m. (n=5-8), ns denotes not significant, P<0.05 (*), P< 0.01 (**), P<0.001 (***).

**Supplemental Figure 4. Flow cytometry gating strategy.** Bronchoalveolar lavage fluid (BALF) was analyzed using flow cytometry, to determine the percentage of alveolar macrophages and neutrophils (from CD45 positive cells) after intranasal LPS (1 µg) administration.

**Supplemental Figure 5. Bacterial growth in liver and Ly-6G in lung upon *K. pneumoniae*** **infection.** Bacterial colony forming units (CFU) of the liver at 12 h and 36 h post infection (**A**). Sections of lung were cut, stained and quantified for Ly-6G (see supplementary methods) (**B**). The abbreviations used to indicate vendors are as follows: Janvier (Jan), Envigo (Env) and Charles River (CR). Data is shown as median (CFU) or mean ± s.e.m. (Ly-6G), n=5-8, ns denotes not significant.

**Supplemental references**

1. Lankelma JM, Birnie E, Weehuizen TAF, et al (2017) The gut microbiota as a modulator of innate immunity during melioidosis. PLoS Negl Trop Dis 11: e0005548.

2. Claushuis TAM, de Vos AF, Nieswandt B, et al (2018) Platelet glycoprotein VI aids in local immunity during pneumonia-derived sepsis caused by gram-negative bacteria. Blood 131: 864-876.

3. Schuijt TJ, Lankelma JM, Scicluna BP, et al (2016) The gut microbiota plays a protective role in the host defence against pneumococcal pneumonia. Gut 65: 575-583.

4. Anas AA, Hovius JW, van 't Veer C, et al (2010) Role of CD14 in a mouse model of acute lung inflammation induced by different lipopolysaccharide chemotypes. PLoS One 5: e10183.

5. Costea PI, Zeller G, Sunagawa S, et al (2017) Towards standards for human fecal sample processing in metagenomic studies. Nat Biotechnol 35: 1069-1076.

6. Kozich JJ, Westcott SL, Baxter NT, et al (2013) Development of a dual-index sequencing strategy and curation pipeline for analyzing amplicon sequence data on the MiSeq Illumina sequencing platform. Appl Environ Microbiol 79: 5112-5120.

7. Callahan BJ, McMurdie PJ, Rosen MJ, et al (2016) DADA2: High-resolution sample inference from Illumina amplicon data. Nat Methods 13: 581-583.

8. Wang Q, Garrity GM, Tiedje JM, et al (2007) Naive Bayesian classifier for rapid assignment of rRNA sequences into the new bacterial taxonomy. Appl Environ Microbiol 73: 5261-5267.

9. Quast C, Pruesse E, Yilmaz P, et al (2012) The SILVA ribosomal RNA gene database project: improved data processing and web-based tools. Nucleic Acids Res 41: D590-D596.

10. McMurdie PJ, Holmes S (2013) phyloseq: an R package for reproducible interactive analysis and graphics of microbiome census data. PLoS One 8: e61217.

11. Lahti L, Shetty S (2017) Tools for microbiome analysis in R. Available at: http://microbiome.github.com/microbiome. Accessed February 13, 2020.

12. de Porto AP, Liu Z, de Beer R, et al (2019) Btk inhibitor ibrutinib reduces inflammatory myeloid cell responses in the lung during murine pneumococcal pneumonia. Mol Med 25: 3.
