# Supplementary figures and images for: Vendor effects on murine gut microbiota and its influence on lipopolysaccharide-induced lung inflammation and Gram-negative pneumonia
Source: Intensive Care Med Exp. 2020 Aug 25;8:47. doi: 10.1186/s40635-020-00336-w (PMC7447702; doi:10.1186/s40635-020-00336-w)

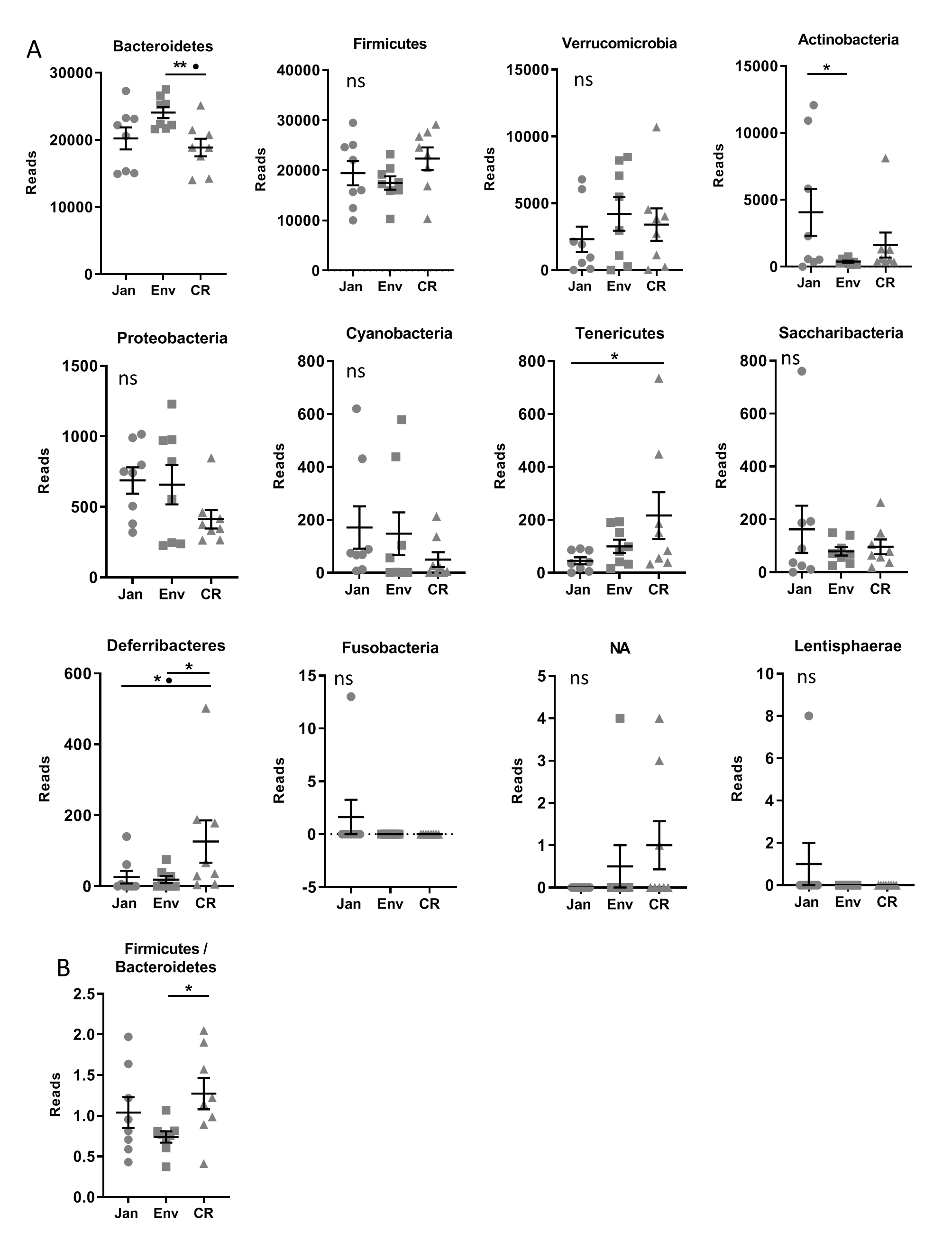

Supplement: Supplementary file 2 — Additional file 2: Supplemental Figure 1. Phylum abundance of gut microbiota between vendors. Graphs show the reads per vendor for each phylum separately, NA denotes non annotatable reads with unknown phylum classification (A), and the ratio of Firmicutes/Bacteroidetes (B). Of note, only Bacteroidetes and Deferribacteres had P<0.05 in the Kruskal-Wallis analysis. The abbreviations used to indicate vendors are as follows: Janvier (Jan), Envigo (Env) and Charles River (CR). Results are shown as mean ± s.e.m. (n= 8), ns denotes not significant, P<0.05 (*), P< 0.01 (**), ● denotes adjusted p-value <0.05 using the Benjamini and Hochberg analysis. [file 40635_2020_336_MOESM2_ESM.tif]

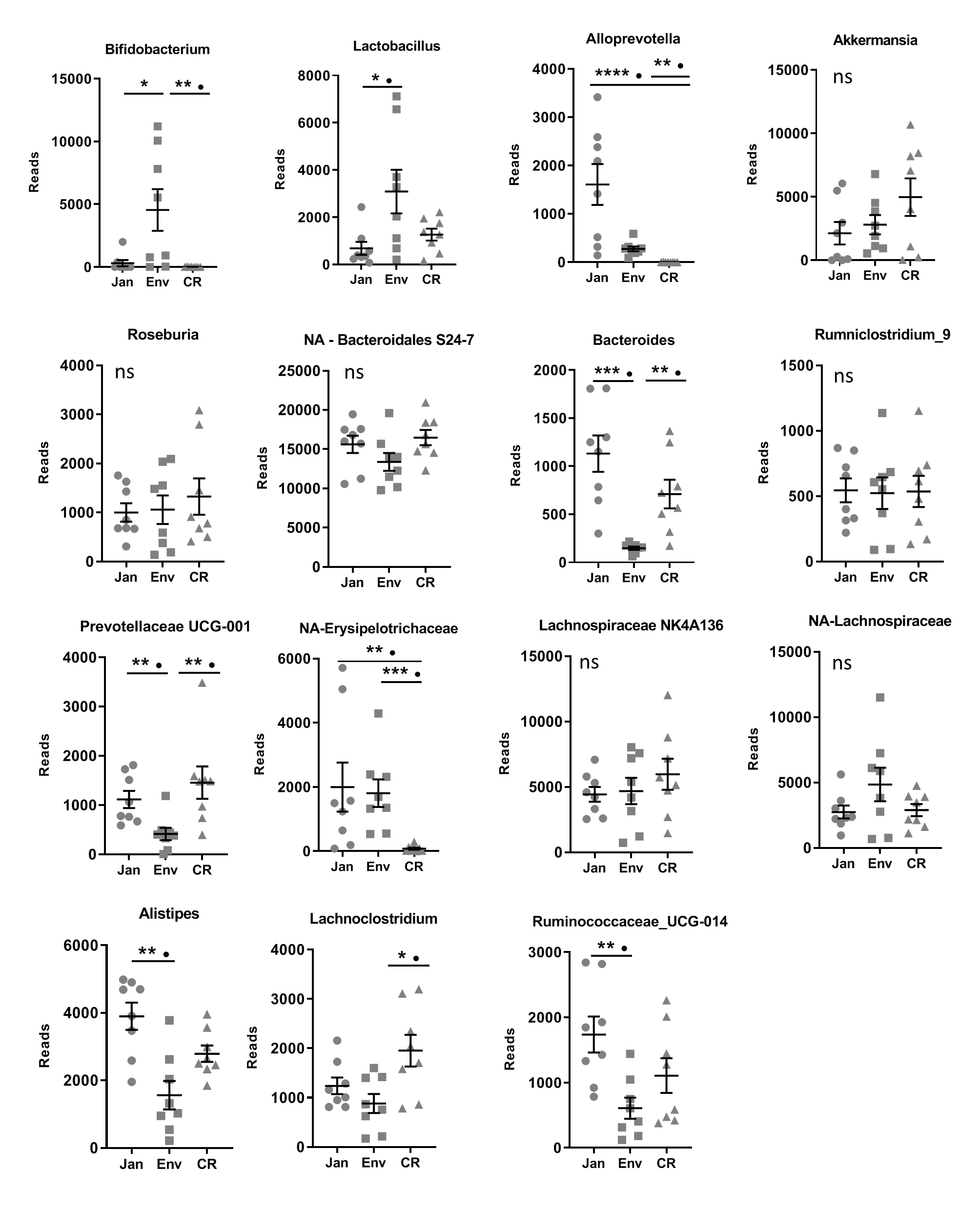

Supplement: Supplementary file 3 — Additional file 3: Supplemental Figure 2. Genera abundance of the top 15 gut microbiota between vendors. Graphs show the reads per vendor for the top 15 genera (based on abundance), one graph per genus. The abbreviations used to indicate vendors are as follows: Janvier (Jan), Envigo (Env) and Charles River (CR). Results are shown as mean ± s.e.m. (n= 8), ns denotes not significant, P<0.05 (*), P< 0.01 (**), P<0.001 (***), P<0.0001 (****), ● denotes adjusted p-value <0.05 using the Benjamini and Hochberg analysis. [file 40635_2020_336_MOESM3_ESM.tif]

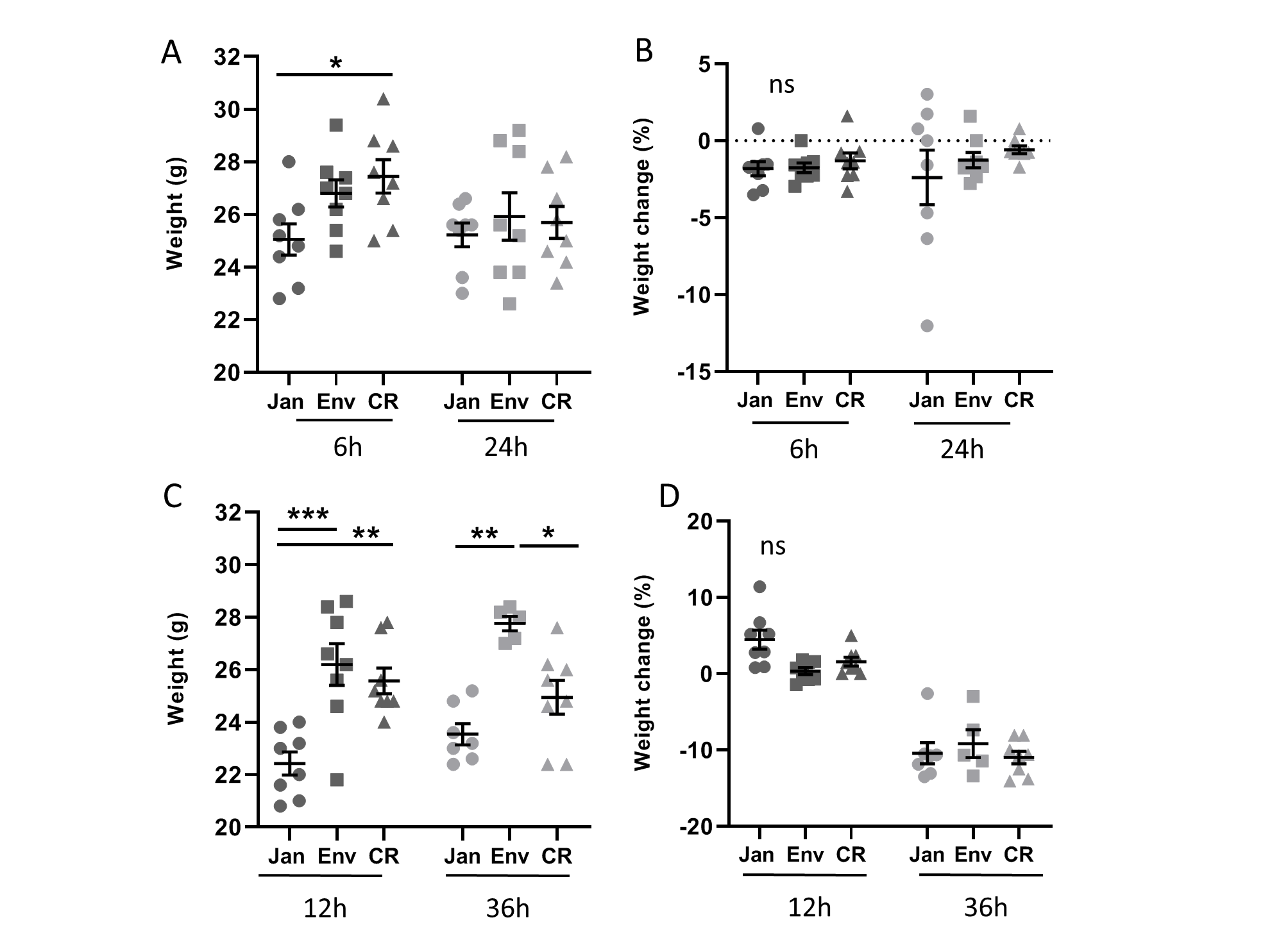

Supplement: Supplementary file 4 — Additional file 4: Supplemental Figure 3. Murine weight prior to administration of LPS or K. pneumoniae and weight change during challenges. Mice were weight prior to inoculation and at sacrifice. The weight prior to administration of K. pneumoniae LPS (A) and K. pneumoniae (C). For these graphs the time points show the groups separated to the time at which they will be sacrificed after the infection/inflammation. The change in weight during K. pneumoniae LPS challenge (B) and K. pneumoniae infection (D). The abbreviations used to indicate vendors are as follows: Janvier (Jan), Envigo (Env) and Charles River (CR). Results are shown as mean ± s.e.m. (n=5-8), ns denotes not significant, P<0.05 (*), P< 0.01 (**), P<0.001 (***). [file 40635_2020_336_MOESM4_ESM.tif]

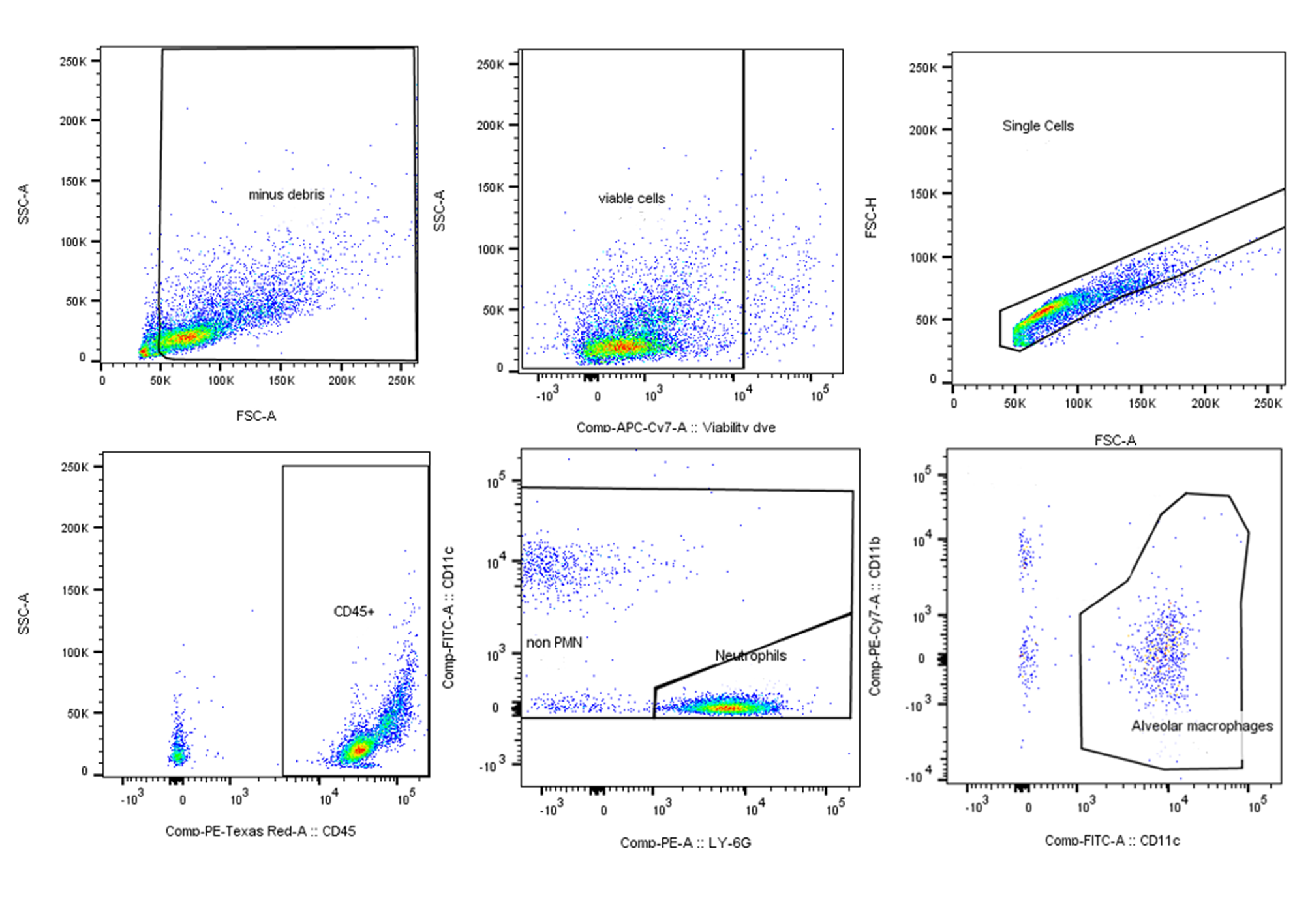

Supplement: Supplementary file 5 — Additional file 5: Supplemental Figure 4. Flow cytometry gating strategy. Bronchoalveolar lavage fluid (BALF) was analyzed using flow cytometry, to determine the percentage of alveolar macrophages and neutrophils (from CD45 positive cells) after intranasal LPS (1 μg) administration. [file 40635_2020_336_MOESM5_ESM.tif]

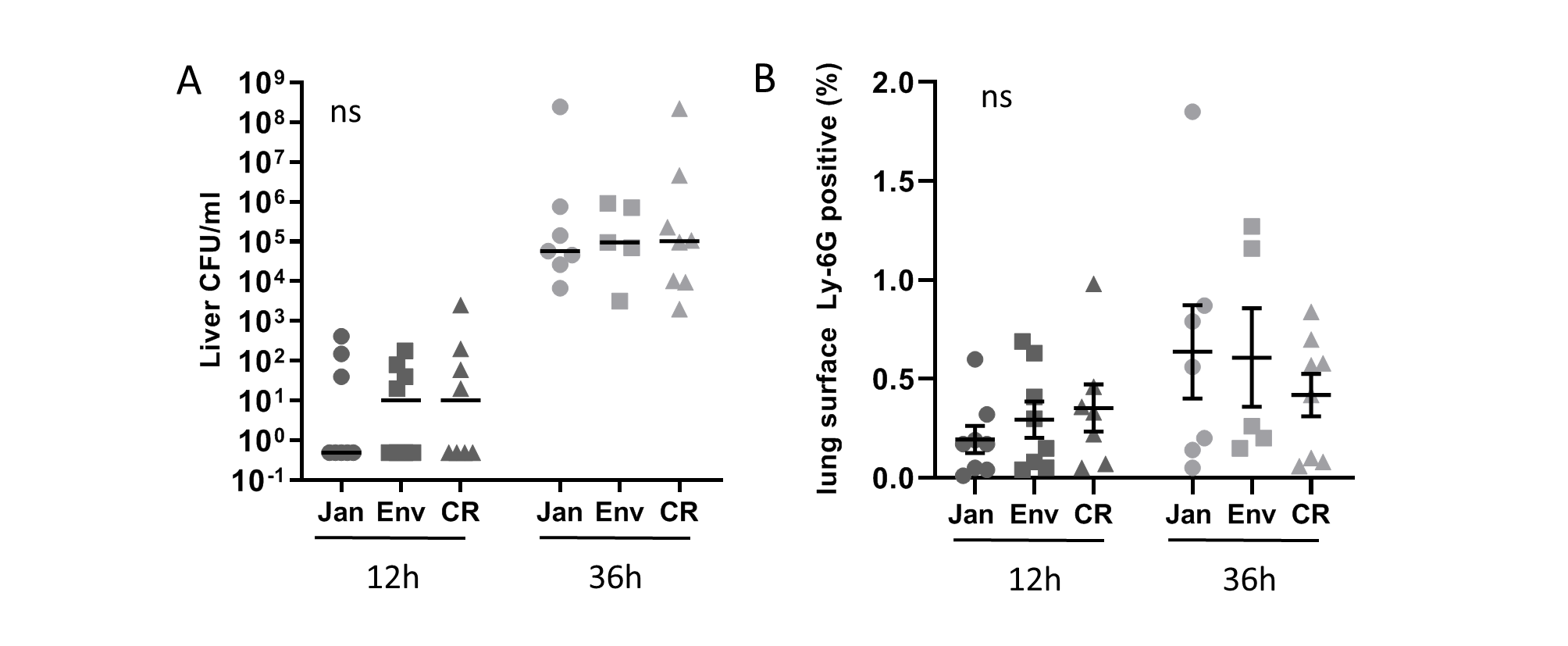

Supplement: Supplementary file 6 — Additional file 6: Supplemental Figure 5. Bacterial growth in liver and Ly-6G in lung upon K. pneumoniae infection. Bacterial colony forming units (CFU) of the liver at 12 h and 36 h post infection (A). Sections of lung were cut, stained and quantified for Ly-6G (see supplementary methods) (B). The abbreviations used to indicate vendors are as follows: Janvier (Jan), Envigo (Env) and Charles River (CR). Data is shown as median (CFU) or mean ± s.e.m. (Ly-6G), n=5-8, ns denotes not significant. [file 40635_2020_336_MOESM6_ESM.tif]
